# Photoelectrocatalytic application of palladium decorated zinc oxide-expanded graphite electrode for the removal of 4-nitrophenol: experimental and computational studies†

**DOI:** 10.1039/c8ra00180d

**Published:** 2018-03-13

**Authors:** Eseoghene H. Umukoro, Moses G. Peleyeju, Azeez O. Idris, Jane C. Ngila, Nonhlangabezo Mabuba, Lydia Rhyman, Ponnadurai Ramasami, Omotayo A. Arotiba

**Affiliations:** Department of Applied Chemistry, University of Johannesburg South Africa oarotiba@uj.ac.za p.ramasami@uom.ac.mu; Centre for Nanomaterials Science Research, University of Johannesburg South Africa; Computational Chemistry Group, Department of Chemistry, Faculty of Science, University of Mauritius Réduit 80837 Mauritius

## Abstract

A novel Pd–ZnO-expanded graphite (EG) photoelectrode was constructed from a Pd–ZnO-EG nanocomposite synthesised by a hydrothermal method and characterised using various techniques such as X-ray diffractometry (XRD), Raman spectroscopy, UV-Vis diffuse reflectance spectroscopy, nitrogen adsorption–desorption analysis, transmission electron microscopy (TEM), scanning electron microscopy (SEM) and energy dispersive spectrometry (EDS). Cyclic voltammetry and photocurrent response measurements were also carried out on the electrode. The Pd–ZnO-EG electrode was employed in the photoelectrocatalytic removal of 4-nitrophenol as a target water pollutant at a neutral pH and with a current density of 7 mA cm^−2^. Optical studies revealed that the Pd–ZnO-EG absorbed strongly in the visible light region. The Pd–ZnO-EG electrode showed improved photoelectrocatalytic activity in relation to ZnO-EG and EG electrodes for the removal of the 4-nitrophenol. The photocurrent responses showed that the Pd–ZnO-EG nanocomposite electrode could be employed as a good photoelectrode for photoelectrocatalytic processes and environmental remediation such as treatment of industrial waste waters. Density functional theory method was used to model the oxidative degradation of 4-nitrophenol by the hydroxyl radical which generates hydroquinone, benzoquinone, 4-nitrocatechol, 4-nitroresorcinol and the opening of the 4-nitrophenol ring. Furthermore, the hydroxyl radical is regenerated and can further oxidise the ring structure and initiate a new degradation process.

## Abstract 2

Band diagram for the mechanism of charge transfer involved in the photo-electrocatalytic degradation activity of Pd-ZnO-expanded graphite (EG) photoanode on 4-nitrophenol removal.

## Graphical abstract

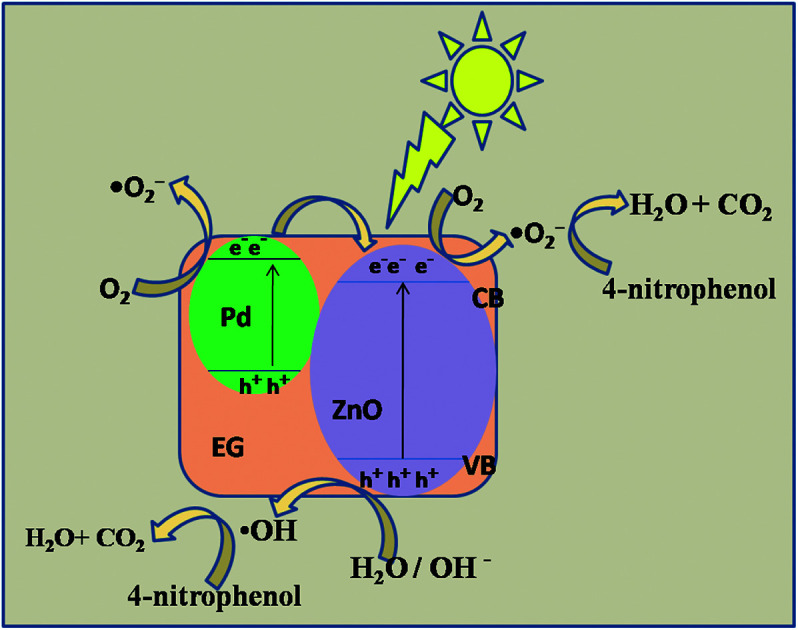

## 1 Introduction

The efficient and effective disposal of organic pollutants in the environment is a necessity for sustainable development of society. However, this is a major challenge due to the growth of industrialisation.^1^ Nitrophenol and its derivatives are organic pollutants which are mostly used in agricultural, textile, paper, explosives and pharmaceutical industries.^2,3^ They are potentially hazardous and mutagenic to humans and the general ecosystem.^2,4^ Among the derivatives of nitrophenols, 4-nitrophenol is found to be highly toxic, stable and biorefactory in nature.^2,4^ Therefore, the removal of 4-nitrophenol from industrial wastewaters is essential. Several traditional methods have been utilised for the treatment of these 4-nitrophenol-containing wastewaters, but the poor removal rate is still a general concern due to its high resistance to biodegradation.^5–8^

In recent years, electrochemical methods have been successfully developed as alternatives to the conventional methods for the removal of organic pollutants. This is mainly due to the generation of highly reactive hydroxyl radical for the non-selective oxidation of these pollutants.^9–11^ This process entails the electrochemical production of hydroxyl radical at the surface of the electrode which is followed by its reaction with the organic pollutants.^9,11^ The advantages of electrocatalytic degradation technique include the immobilisation of the catalyst (electrode) which helps in minimising catalyst separation from the reaction system, controlled reaction conditions and the relatively low equipment cost.^10,12^ However, some drawbacks have also been reported such as the oxygen evolution which takes place simultaneously with the oxidation of the pollutant resulting from the application of high voltage, the low concentration of the pollutant raises the problem of mass transfer and the processes can be time consuming.^10,13,14^ Thus, it is important to improve this technique by the concurrent use of visible light irradiation (photolysis), leading to a process known as photoelectrocatalysis. In this process, a photoanode is constructed by mounting a semiconductor material that is photoactive on a conducting support with the simultaneous application of light and a bias voltage. This leads to the production of non-selective oxidants such as hydroxyl radical by the semiconductor and the conducting support for the degradation of the pollutants.^9,15^ TiO_2_ semiconductor photoanode is the most widely used semiconductor photoanode.^14–20^ Nevertheless, in recent years, ZnO is being utilised as an alternative to TiO_2_ due to the nontoxicity, availability, low cost, chemical and thermal stability, good catalytic efficiency and optoelectronic nature of ZnO.^21–24^ ZnO has also found applications in photovoltaic devices, water splitting and organic pollutants degradation.^25,26^ However, the fundamental problem of ZnO is the recombination rate of the photoexcited holes and electrons which hinders its photocatalytic efficiency. Generally, the kinetics of the recombination rate is faster than that of the surface reduction–oxidation reactions, and this results in a decrease of the quantum efficiency of photocatalysis.^23,24^ Different techniques have been used to reduce the electron–hole recombination rate and these include the introduction of semiconductor oxides, carbonaceous materials and noble metals.^23,24,27^ Noble metals nanoparticles such as Au, Ag, Pt and Pd have been reported to enhance the photocatalytic activity of ZnO.^28–30^ In comparison with Pt and Au, Pd is cheaper and it has been employed in catalytic processes.^24^ Pd nanoparticles can act as collectors for the photoexcited electrons and these aid the charge transfer and transportation processes, thereby creating more active sites for the catalytic reaction.^31^ The use of expanded graphite (EG) can also help in reducing the recombination rate of ZnO. EG is a carbonaceous material consisting of two dimensional bonded carbons which possesses excellent electrical conductivity, large surface area, good porosity, compressibility, low density and excellent electron transfer and transport properties. In addition, it possesses good mechanical and thermal stability, good resistance to high temperature, and can withstand high voltage.^15,32–34^ The inclusion of EG in Pd–ZnO-EG can result in the EG acting as an electron sink for photoexcited electrons from ZnO due to the unique electron transport capability of EG.^19^ As a result, the problem of electron–hole recombination rate of ZnO would be minimised. Thus, the photoactivity of ZnO in the Pd–ZnO-EG nanocomposite would be enhanced by forming more oxidants such as hydroxyl radical for the degradation of organic pollutants. The EG can also be used as a conducting support for the immobilisation and trapping of the ZnO and Pd, therefore, minimising the losses from the recovery of these photocatalysts.^15^

Considering the unique properties of Pd, ZnO and EG, it can be hypothesised that Pd–ZnO-EG nanocomposite can act as a promising photoeletrocatalytic material for the degradation of 4-nitrophenol. In this novel study, Pd–ZnO supported on EG was synthesised, fabricated into a photoanode and used for the photoelectrocatalytic removal of 4-nitrophenol as a model organic pollutant. Of importance is the understanding of the degradation pattern and products during the photoelectrocatalytic process because it can be used to estimate the toxicity of the product in comparison with the initial organic pollutants, the time of reaction, the kinetics and mechanism of reaction. To better understand the degradation mechanism, we have employed density functional theory (DFT) method to predict the degradation products and the different possible degradation pathways for the 4-nitrophenol degradation which is in agreement with experimental findings reported in literature.

## 2 Experimental section

### 2.1 Chemicals

Nitric acid, sulphuric acid, natural graphite, sodium sulphate, zinc acetate dehydrate, potassium hexacyanoferrate(ii), sodium hydroxide, potassium chloride, 4-nitrophenol and potassium hexacyanoferrate(iii). These were bought from Sigma Aldrich, Germany and used for the experiment without further purification.

### 2.2 Synthesis of expanded graphite (EG)

The expanded graphite was prepared by employing our previous technique.^35,36^ Sifted flakes of natural graphite (300 μm) were immersed in a 1 : 3 (vol/vol) mixture of concentrated nitric and sulphuric acids for 48 h at ambient conditions for the intercalation of the graphite material. The intercalated graphitic material was washed properly to achieve a pH value close to 7, and dried in the air. Then, the expansion of the graphitic material was done by heating the material at about 800 °C to form a puffed-up material called expanded graphite.

### 2.3 Synthesis and construction of Pd–ZnO-EG nanocomposite

Zinc acetate dehydrate (3.29 g), 2.4 g of sodium hydroxide and 2 g of the prepared EG were mixed in 70 mL of ethanol solution and stirred for 30 min. The mixture was poured into a Teflon-lined autoclave and thermally treated at 160 °C for 24 h. The autoclave was left to cool to ambient temperature. The ZnO-EG formed was centrifuged, washed several times with water and ethanol, and dried overnight at 60 °C in an oven. In order to prepare the Pd–ZnO-EG, a certain amount of the ZnO-EG was weighed and dispersed in ethanol, followed by ultrasonication for 30 min. Palladium(ii) acetate in ethanol solution was added and ultrasonicated further for 20 min. The resulting mixture was put in a water bath and kept at 70 °C for 3 h. The mixture was filtered and dried at 60 °C overnight. The amount of Pd in the composite was 5% by weight of the Pd–ZnO-EG nanocomposite.

### 2.4 Construction of Pd–ZnO-EG, ZnO-EG and EG electrodes

The construction of the electrodes was done using a technique described in our previous studies.^11,15,19,37^ The as-prepared EG, ZnO-EG and Pd–ZnO-EG nanocomposites were used to make pellets using a hydraulic press at a pressure of about 10 000 psi. These pellets were utilised for fabricating the electrodes. Copper wire, conductive silver paint and epoxy sealer were also used. These pellets were placed on the coiled copper wire with the assistance of the conductive silver paint. This was left out in ambient air overnight. The pellets edges were then sealed with the epoxy sealer in order for electricity to flow only from the basal plane. This was placed in a glass rod before further use.

### 2.5 Characterisation of EG, ZnO-EG and Pd–ZnO-EG nanocomposites

X-ray diffractometer (Philips PAN Analytical X'Pert powder, Netherlands) with Cu-Kα radiation (*λ* = 0.15418) was used to determine the crystallinity of the as-prepared materials. The Raman spectra were measured with Witec alpha300 R confocal Raman microscope (Germany). The spectra from the UV absorbance were done at a wavelength of 200–800 nm with Shimadzu UV-2450 (Japan). Morphological images of the as-prepared samples were taken with scanning electron microscopy (TESCAN, VEGA 3 XMU, Czech Republic) and transmission electron microscopy (JEOL JEM-2100, USA) at 200 kV accelerating voltage. Energy-dispersive X-ray spectrometer (TESCAN, Czech Republic) connected to the SEM was employed in measuring the samples' elemental composition. The extent of mineralisation was measured with Teledyne Tekmar TOC Analyzer (USA). Cary 60 UV-Visible spectrophotometer (Agilent technologies, USA) was utilised in determining the removal efficiency of the 4-nitrophenol. The source of visible light irradiation was Oriel LCA-100 Solar Simulator (USA) which was equipped with 100 W xenon lamp and AM1.5G filter.

### 2.6 Photoelectrochemical measurements

Electrochemical and photoelectrochemical behaviours of the EG, ZnO-EG and Pd–ZnO-EG nanocomposite electrodes were investigated in a redox probe of 5 mM of potassium ferrocyanide and ferricyanide in 0.1 M KCl solution. Cyclic voltammetry (CV) and chronoamperometry photocurrent responses under visible light (Oriel LCA-100 Solar Simulator) were investigated in a three-electrode electrochemical configuration with Autolab 302N potentiostat (The Netherlands), while the working electrodes (with diameter of 1.3 cm and geometric area of 1.33 cm^2^) were the as-prepared materials, platinum foil was used as counter electrode and the reference electrode was Ag/AgCl (3.0 M KCl).

### 2.7 Electrochemical and photoelectrocatalytic experiments

The photoelectrocatalytic activity of the materials were determined by the degradation of 4-nitrophenol. This was carried out in a reactor (100 mL) with 20 ppm of 4-nitrophenol. A solution of 0.1 M Na_2_SO_4_ was used as supporting electrolyte. The electrodes were positioned to face the irradiation from the solar simulator. The source of power was the potentiostat, while Oriel LCA-100 Solar Simulator was the source of irradiation. In order to achieve a power beam of 1 sun which is equivalent to a 100 mW cm^−2^, an AM1.5G filter was placed in the simulator and the reactor was 8 cm away from the irradiation source. A bias potential was applied without making use of the visible light for the electrocatalytic removal of the 4-nitrophenol. At 25 min intervals, an aliquot of 4-nitrophenol was withdrawn from the reactor with disposable syringe over a period of 2 h 30 min. The aliquot was then filtered and the removal efficiency of the 4-nitrophenol was measured on a UV-Visible spectrophotometer. The total organic content was investigated on a TOC analyser (Teledyne Tekmar TOC fusion). Furthermore, the influence of pH, current density and percentage of Pd in the nanocomposite were studied.

### 2.8 Computational details

Quantum chemical calculations based on density functional theory (DFT) method were used to study the different possible pathways for the degradation of 4-nitrophenol. Full geometry optimisations were carried out in the gas phase with the B3LYP^38,39^ functional with the 6-31G(d) basis set.^40^ The optimisations were followed by frequency computations at the same level of theory to confirm that the stationary points are minima (no imaginary frequencies) and that each of the transition state (TS) structures has only one imaginary frequency. The reaction pathways were subjected to intrinsic reaction coordinate (IRC)^41,42^ analysis in order to trace their paths and to confirm that the optimised TS connects the correct reactant and product. Zero-point energy (ZPE) correction is included in all the reported electronic energies. All the computations were carried out using the Gaussian 09 software suite^43^ running on the GridChem Science Gateway.^44–46^

## 3 Results and discussion

### 3.1 Raman studies

**Fig. 1:**
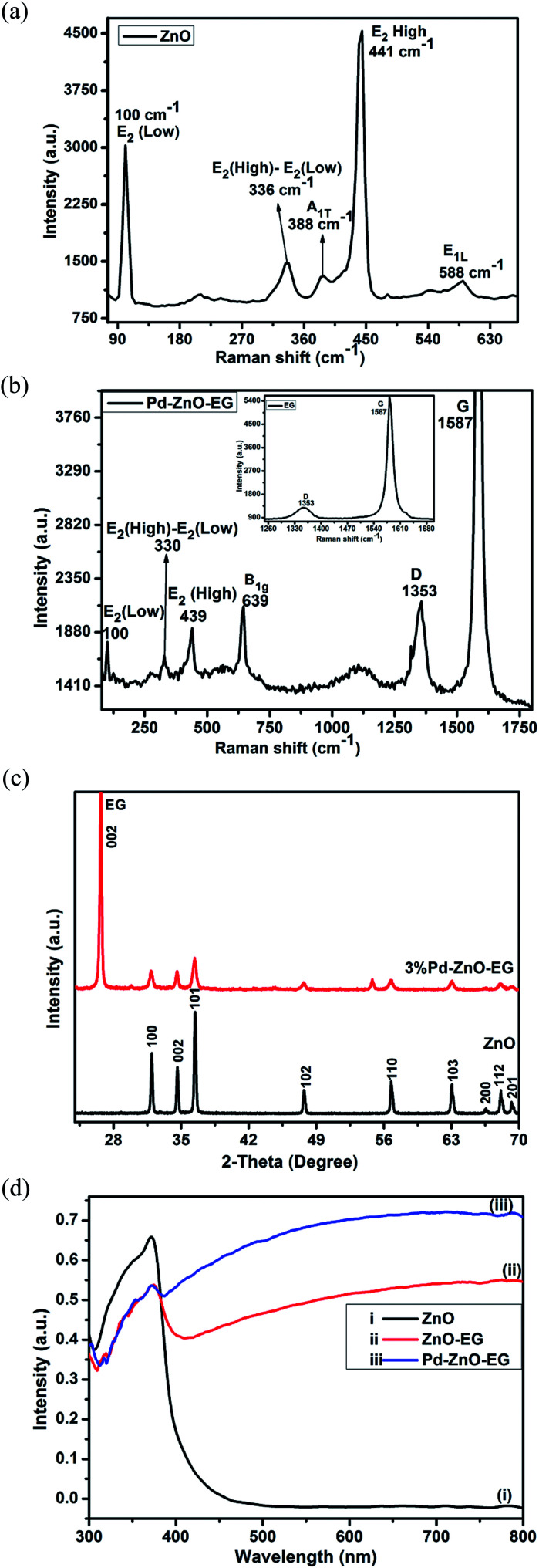
Raman spectra of (a) ZnO and (b) Pd–ZnO-EG and EG (inset); (c) X-ray diffractograms of ZnO and 3% Pd–ZnO-EG; (d) UV-Visible absorbance spectra of (i) ZnO, (ii) ZnO-EG and (iii) Pd–ZnO-EG.

The Raman spectra of the EG and Pd–ZnO-EG materials are illustrated in Fig. 1a and b. The band observed at 100 cm^−1^ of the Raman spectrum of ZnO (Fig. 1a) corresponds to the E_2_ (Low) mode while the peak at 336 cm^−1^ results from the Raman active frequency phonons, E_2_ (High) − E_2_ (Low). The peak around 388 cm^−1^ can be assigned to the A_1T_ mode, while the low intensity peak at 588 cm^−1^ can be ascribed to the E_1L_ mode. In addition, the very intense and characteristic peak at 441 cm^−1^ is attributed to E_2_ (high), considered as the Raman active optical phonon mode. This is an indication that the as-prepared ZnO possesses a wurtzite hexagonal phase.^47–49^ The peak at 639 cm^−1^ is exhibited by Pd nanoparticles which is a Raman active vibrational mode and characteristic feature of PdO.^50,51^ In Fig. 1b, it is clear that additional new peaks appeared near 1353 and 1587 cm^−1^ which are the D and G bands of the EG, respectively (inset). These are due to the in-plane phonon vibration of the sp^2^-bonded carbon atoms of the graphite material (G band) and the defects produced in the graphite sheets (D band) due to the formation of sp^3^ carbon bonding caused by the oxygen groups present in the EG. However, a disappearance of the two peaks at 388 and 588 cm^−1^ was observed. In addition, shifts were noticed in the peaks at 330 and 439 cm^−1^ and this could be due to the incorporation of the Pd and EG.^52,53^ This indicates the successful preparation of the Pd–ZnO-EG nanocomposite.

### 3.2 XRD analysis

The crystallinity and phase of the samples were examined by conducting XRD analysis. The XRD patterns of ZnO and Pd–ZnO-EG are presented in Fig. 1c. The XRD pattern of ZnO shows peaks at 2*θ* = 31.8°, 34.6°, 36.4°, 47.7°, 56.7°, 63.0°, 66.5° and 68.1° which correspond to the crystal plane of (100), (002), (101), (102), (110), (103), (200) and (112). These confirm that the hexagonal wurtzite structure of ZnO was prepared (JCPDS Card no. 36-1451). The conspicuous peak observed at 2*θ* = 26.46° is allocated to the (002) crystal plane of EG, confirming the presence of EG. However, due to the small amount of Pd added, the peaks for Pd were not observed.^24^

### 3.3 UV-visible analysis

The optical behaviour of the materials was determined using UV-Visible analysis. The ZnO-EG exhibited a better photo-absorbance than the ZnO in the region of visible light from *λ* = 430–800 nm (Fig. 1d). This can be attributed to the presence of EG which is photosensitizing in nature.^54^ It can also be observed that Pd–ZnO-EG displayed a better photo-response in the visible light region with a higher intensity relative to the as-prepared ZnO-EG. Firstly, this better photoresponse is an indication of the addition of Pd to the ZnO-EG material. Secondly, the decoration of the ZnO-EG with Pd aided in effective separation and transport of the photogenerated charge carriers.^55^ In the same vein, the surface plasmon resonance behaviour of Pd played a role in improving the light absorption.^56^ It can be deduced that the photosensitizing nature and charge transfer ability of the EG as well as the surface plasmon resonance behaviour of the Pd resulted in a synergic enhancement of the photoactivity observed in the Pd–ZnO-EG nanocomposite.^15,55^

### 3.4 Brunauer–Emmett–Teller (BET) analysis

The determination of the surface area, pore volume and pore size of the ZnO and Pd–ZnO-EG was carried out with the aid of nitrogen adsorption–desorption (BET) analysis. Table 1 shows the obtained results from the analysis. The nitrogen adsorption–desorption isotherms of the prepared Pd–ZnO-EG sample are presented (ESI Fig S1†). It was observed that the Pd–ZnO-EG displayed bigger pore size, pore volume and BET surface area in comparison with the bare ZnO. This is an indication that the incorporation of the Pd and EG led to an increase in surface area which can improve the photoelectrocatalytic degradation of the 4-nitrophenol by the Pd–ZnO-EG electrode.

**Table 1:** BET surface area, pore size and pore volume of the bare ZnO and Pd–ZnO-EG samples

Material	BET surface area (m^2^ g^−1^)	Pore volume (cm^3^ g^−1^)	Pore size (nm)
ZnO	3.2959	0.0105	12.7215
Pd–ZnO-EG	4.9329	0.0354	28.6602

### 3.5 Morphological studies

**Fig. 2:**
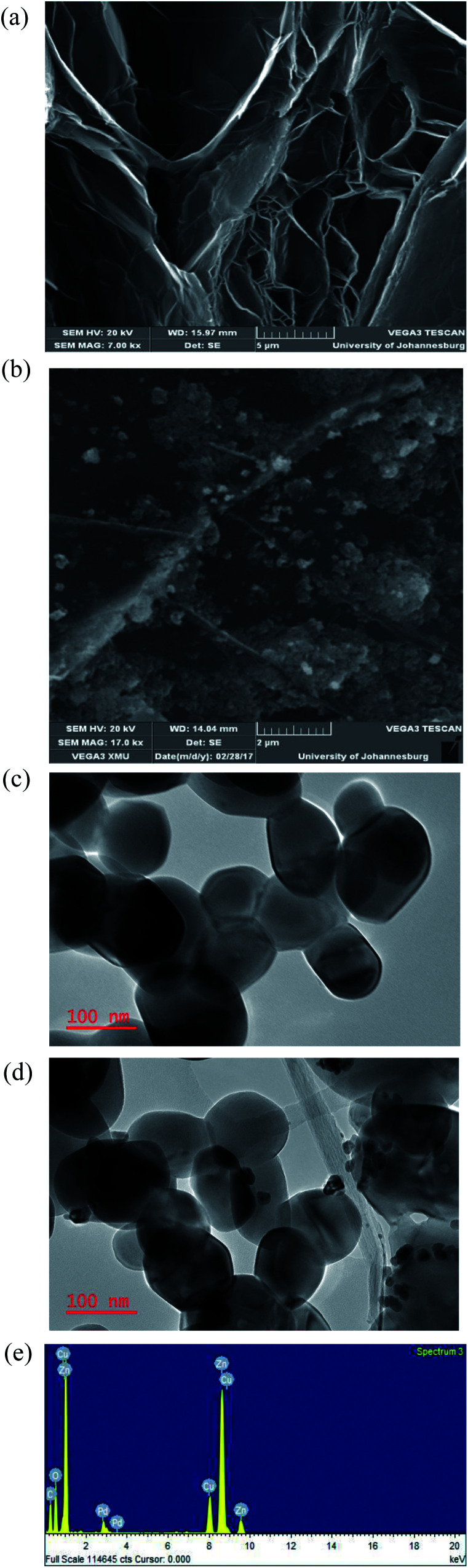
SEM images of (a) expanded graphite, (b) Pd–ZnO nanoparticles trapped in EG sheets. TEM images of (c) ZnO and (d) Pd nanoparticles anchored on ZnO immobilised on the EG. (e) EDX spectra showing the presence of Pd, Zn, O and C in the Pd–ZnO-EG samples.

The morphological images of the as-prepared ZnO, EG, and Pd–ZnO-EG were taken on SEM and TEM, while the elemental composition were determined using an energy dispersive X-ray spectroscopy joined to the TEM. Fig. 2a shows the different layers of graphitic sheets and pores of the EG for the immobilisation of the Pd and ZnO nanoparticles. In Fig. 2b, the ZnO nanoparticles are supported within the layers of the EG sheets. Fig. 2c illustrates the TEM image of the ZnO particles. The scale shows that most of the particles are in the nano regime. Fig. 2d depicts the TEM image of Pd–ZnO-EG where all the three components can be seen. The Pd nanoparticles (smaller nanoparticles) are distributed on the ZnO nanoparticles (larger particles) within the graphitic sheets/layers of the EG. In Fig. 2e, the EDS spectrum shows the elemental composition of the Pd–ZnO-EG to be C, O, Zn and Pd, confirming the successful preparation of the Pd–ZnO-EG nanocomposite.^24^

### 3.6 Photoelectrochemical characterisation

**Fig. 3:**
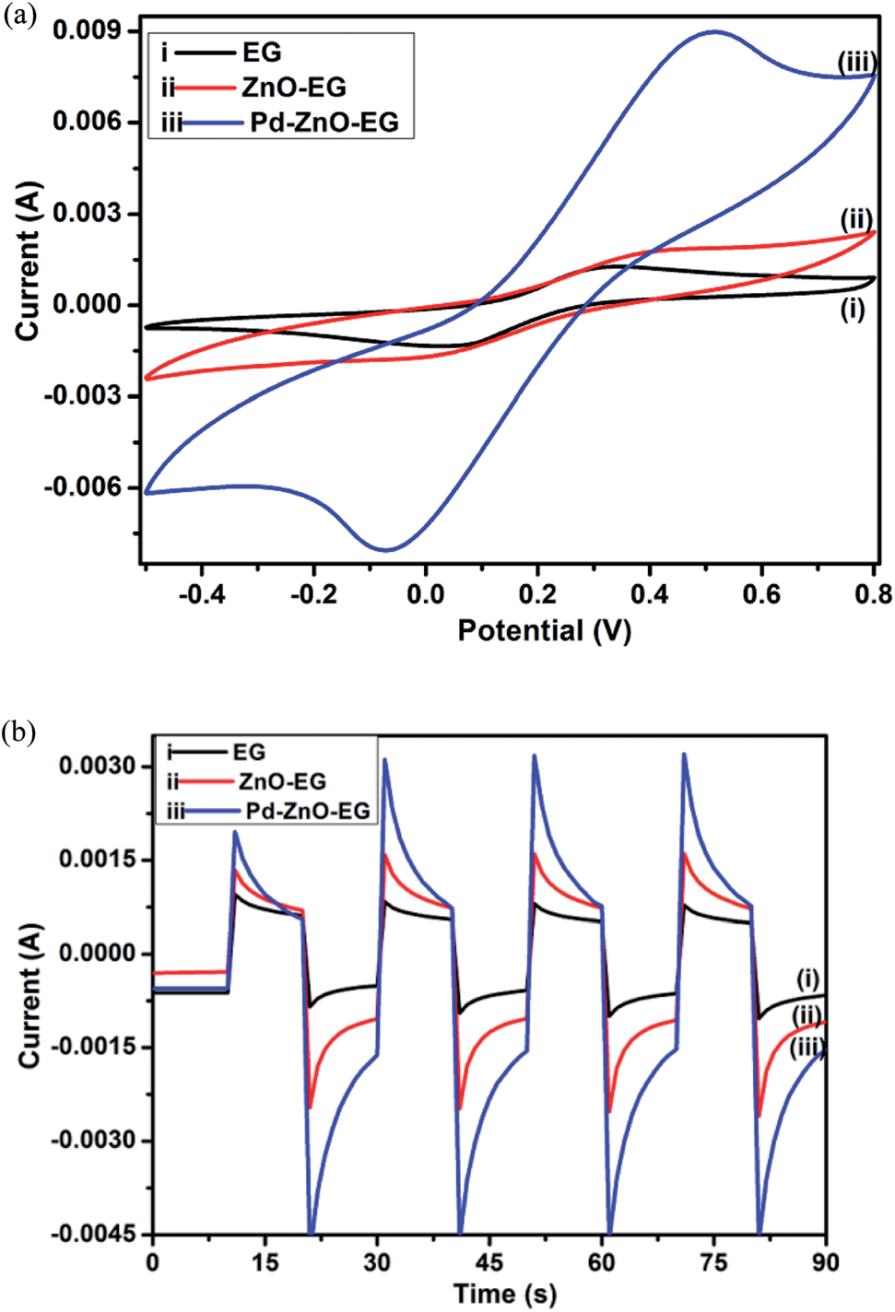
(a) Cyclic voltammograms of (i) EG, (ii) ZnO-EG and (iii) Pd–ZnO-EG electrodes in 5 mM [Fe(CN)_6_]^−3/−4^, 0.1 M KCl solution, at 50 mV s^−1^ scan rate (b) photocurrent responses of (i) EG, (ii) ZnO-EG and (iii) Pd–ZnO-EG electrodes in the dark and under light.

The cyclic voltammetry of the Pd–ZnO-EG displayed a much higher current in relation to EG and ZnO-EG electrodes (Fig. 3a). Lower faradaic currents of the [Fe(CN)_6_]^−3/−4^ were observed from the EG and ZnO-EG when compared to that generated from Pd–ZnO-EG. This may be due to the catalytic property of the Pd nanoparticles which were added to the ZnO-EG to produce the Pd–ZnO-EG material.^24^ The inclusion of Pd increased the electroactive surface area of the electrode, thus the Pd–ZnO-EG is seen to exhibit a higher rate of electrode reaction.^24,35,55^ To support this argument, the electroactive surface areas of the electrodes were calculated using the Randles–Sevcik equation;

1 *i*_p_ = *kn*^3/2^*AD*^1/2^*v*^1/2^*C*

where *D*, diffusion co-efficient, is 7.6 × 10^−6^ for ferrocyanide, *k* is 2.69 is 10^5^, *A* is the area of the electrode active surface, *n* is the number of electrons involved in the reaction process, *C* is concentration of the electrochemical probe and *v* is the scan rate.^57,58^ The calculated electroactive surface areas of the EG, ZnO-EG and Pd–ZnO-EG electrodes are 1.60, 2.27 and 10.91 mm^2^ respectively. These suggest that there was a considerable increase in the electrochemical active sites after the addition of the Pd nanoparticle.^35^ Chronoamperometry technique was employed to study the photocurrent responses of the EG, ZnO-EG and Pd–ZnO-EG materials. The efficiency of charge carriers separation and transfer is proportional to the photocurrent response of the material.^17,59,60^ It is observed from Fig. 3b that the Pd–ZnO-EG exhibited a stronger photoresponse in comparison to the ZnO-EG and EG materials. This suggests that the Pd nanoparticles could effectively retard the recombination of the photo-generated electrons and holes, and thereby improving the photoelectrocatalytic activity of the Pd–ZnO-EG, thus, making it a promising photoelectrode for photoelectrocatalytic processes especially in water treatment process.

### 3.7 Photoelectrocatalytic experiments

**Fig. 4:**
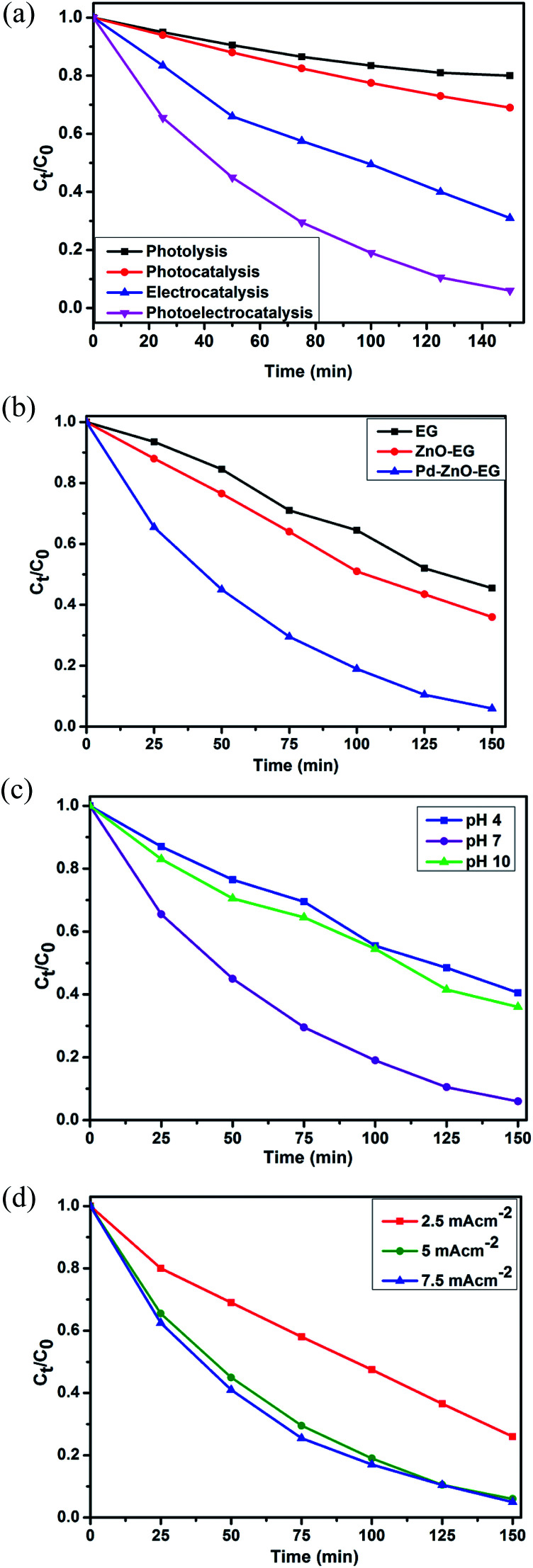
Kinetics curves of (a) electrochemical and photoelectrocatalytic removal of 4-nitrophenol at Pd–ZnO-EG electrode. (b) Photoelectrocatalytic removal of 4-nitrophenol at EG, ZnO-EG and Pd–ZnO-EG electrodes. Effect of various (c) pH conditions and (d) different current densities at Pd–ZnO-EG electrode.

The photoelectrocatalytic activities of the EG, ZnO-EG and Pd–ZnO-EG electrodes were evaluated by the rate of removal of 4-nitrophenol as a target organic pollutant. The removal rate was monitored using UV-Visible spectroscopy and total organic carbon (TOC) analysis. For control experiments, photolytic, photocatalytic and electrocatalytic removal of 4-nitrophenol were carried out and compared with the photoelectrocatalytic process using the Pd–ZnO-EG material.^61^ The results revealed that the removal of 4-nitrophenol was 20% for photolysis, 31% for photocatalysis, 69% for electrocatalysis and 94% for the photoelectrocatalytic process (Fig. 4a). Furthermore, the EG and ZnO-EG electrodes were used as control experiments for the photoelectrocatalytic removal of 4-nitrophenol. As presented in Fig. 4b, the kinetics curves show that the ZnO-EG electrode gave a better removal efficiency compared to that of the EG electrode. This is attributed to the photoactive nature of ZnO in the ZnO-EG material. Moreover, after the addition of Pd to the ZnO-EG material, an enhanced photoelectrocatalytic removal performance (94%) was found. The improved removal efficiency is as a result of the photoactive nature of ZnO and the enhanced absorption of visible light caused by the surface plasma resonance ability of Pd in the composite. The Pd can also behave as electron sink for the photoinduced electrons, thereby minimising the recombination of the electron–hole pairs generated.^24,62^ In addition, the ability of the EG to act as electron sink as well as its electron transport nature must have aided in inhibiting the recombination of the photoinduced holes and electrons which led to the improved photoelectrocatalytic performance of the Pd–ZnO-EG electrode. The synergic combination of ZnO, EG and Pd that led to improved photocatalytic degradation can be buttressed with photocurrent responses in Fig. 3b which was highest for the Pd–ZnO-EG electrode. The extent of mineralisation of the 4-nitrophenol was evaluated on a TOC analyser after a period of 150 min. The TOC results showed a removal percentage of 58% for Pd–ZnO-EG electrode which is better in comparison with that of EG (32%) and ZnO-EG (40%) electrodes.

Optimisation of the reaction conditions was carried out by evaluating the influence of change in pH, and current density. The photoelectrocatalytic removal of the 4-nitrophenol using Pd–ZnO-EG was found to be pH dependent as presented in the kinetics curves in Fig. 4c. The results obtained at pH 4, 7 and 10 are 60, 94 and 64%, respectively. With a pKa of 7.2, 4-nitrophenol is expected to be neutral at pH 7. The highest degradation rate obtained from pH 7 may be as a result of the prevailing neutral 4-nitrophenol which is not poised to or less likely to compete with the OH radical. The influence of current density, which is generally proportional to the production of hydroxyl radical,^63^ on the removal efficiency of 4-nitrophenol on the Pd–ZnO-EG electrode was investigated (Fig. 4d). As the current density was increased from 2.5 to 5 mA cm^−2^, the removal efficiency increased from 73 to 94%. However, no significant increase in the removal efficiency was observed when the current density was increased to 7.5 mA cm^−2^. This could be attributed to increase in oxygen evolution which tends to compete with the oxidation of the organic pollutant.^63^ The production of intermediates during the degradation process can constitute a film on the surface of the electrode and reduce its activity. Consequently, increasing the current density further from 5 to 7.5 mA cm^−2^ would not lead to effectiveness of cost and energy for the photoelectrocatalytic removal of the 4-nitrophenol since there is no large difference in the increase in the removal of 4-nitrophenol at a current density of 7.5 mA cm^−2^. Thus, a current density of 5 mA cm^−2^ was employed for the experiments for energy efficiency.

### 3.8 Photoelectrocatalytic degradation kinetics and mechanism

The rate of reaction of the degradation of 4-nitrophenol at the EG, ZnO-EG and Pd–ZnO-EG electrode were investigated by modelling the obtained data with Langmuir–Hinshelwood first-order kinetics equation;

2 *r* = d*C*/d*t* = *k*_r_*K*_ad_*C*/(1 + *K*_ad_*C*)

where *r* is the degradation rate, *C* is the concentration of 4-nitrophenol at a particular time, *t*, *K*_ad_ is the reactant coefficient of adsorption, and *k*_r_ is the rate constant of reaction. At very low initial concentration and relatively weak adsorption, *K*_ad_*C* is ≪ 1, and the equation can be simplified to apparent first-order kinetic having *k*_app_ as the apparent first-order rate constant;

3 ln *C*_0_/*C*_*t*_ = *k*_r_*K*_ad_*t* = *k*_app_*t*

**Fig. 5:**
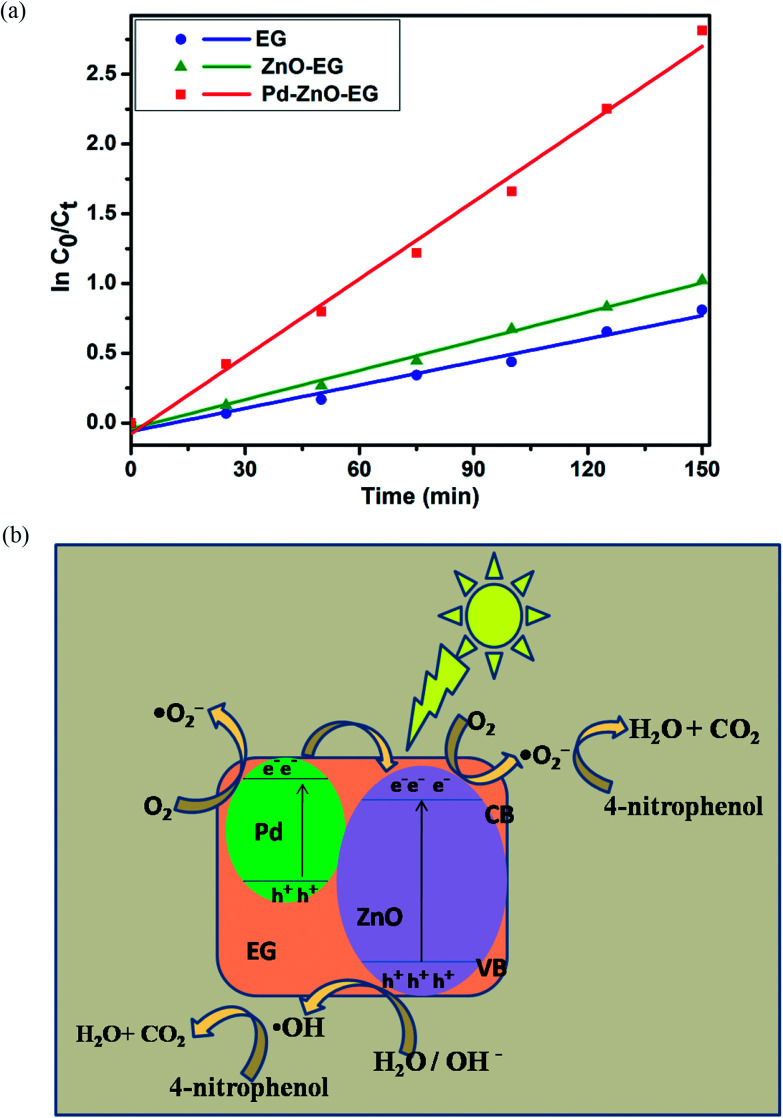
(a) Removal kinetics plots of EG, ZnO-EG and Pd–ZnO-EG for the photo-electrocatalytic removal of 4-nitrophenol at neutral pH and 7 mA cm^−2^ at Pd–ZnO-EG electrode. (b) Proposed mechanism for the charge transfer involved in the photo-electrocatalytic activity of Pd–ZnO-EG electrode for the removal of 4-nitrophenol.

*C*_0_ is the 4-nitrophenol initial concentration, *C*_*t*_ is the its concentration at reaction time, *t*. Fig. 5a depicts that the ln *C*_0_/*C*_*t*_*versus* time curves of the obtained data fitted to give linear kinetics curves with coefficients of correlation greater than 0.95. This implies that reaction for the photoelectrocatalytic removal of 4-nitrophenol follows the pseudo first-order reaction kinetics model, and it can be seen that the Pd–ZnO-EG electrode displayed a better rate of degradation in comparison to EG and ZnO-EG electrodes. The estimated rate constants and correlation coefficients for the electrodes are 5.53 × 10^−3^ min^−1^ and 0.9753 for EG; 6.97 × 10^−3^ min^−1^ and 0.9928 for ZnO-EG; and 18.52 × 10^−3^ min^−1^ and 0.9913 for Pd–ZnO-EG.

A plausible mechanism was proposed to describe the photoelectrocatalytic process and the reactions taking place for the 4-nitrophenol removal are presented in eqn (4)–(9);

4 *hv* + ZnO → ZnO (h_VB_^+^ + e_CB_^−^)

5 e^−^ + O_2_ → ˙O_2_^−^

6 h^+^ + H_2_O → H^+^ + ˙OH

7 ˙O_2_^−^ + 2H^+^ → ˙OH + ˙OH

8 ˙OH + 4-nitrophenol → intermediates → CO_2_ + H_2_O

9 ˙O_2_^−^ + 4-nitrophenol → intermediates → CO_2_ + H_2_O

The difference in the photoelectrocatalytic performance of the different electrodes can be explained using the difference in the work function of ZnO (5.2 eV) and Pd (5.12 eV). It is generally known that a Schottky junction is formed when two materials having different work functions are connected. Electrons flow from the low work function material to the one with a higher work function.^64,65^ Since the Fermi energy of Pd metal (lower work function) is higher than the Fermi level of ZnO semiconductor (higher work function), electrons tend to transfer from the Pd to the ZnO (Fig. 5b) until a new Fermi energy level is achieved at equilibrium. At this new Fermi energy level, an Ohmic-type junction which is a metal-semiconductor Schottky barrier is formed under equilibrium.^24,64,65^ Upon irradiation with visible light, electrons and holes are photoexcited in the ZnO and the photoinduced electrons in the conduction band of the ZnO are transferred to the Pd under the influence of the static electric field at the Schottky junction,^24,65^ thus, an effective charge separation is facilitated. The separated and the remaining electrons on the ZnO conduction band react with dissolved oxygen molecules to generate superoxide radicals which react with water molecules to give hydroxyl radicals. The photoinduced holes can migrate to the surface of the ZnO and react with water on the semiconductor surface to form hydroxyl radicals. Based on eqn (7)–(9), these generated radicals further react with the 4-nitrophenol pollutant to give intermediates which undergo mineralisation to produce CO_2_ and H_2_O.

### 3.9 Computational modelling of the degradation of 4-nitrophenol

**Fig. 6:**
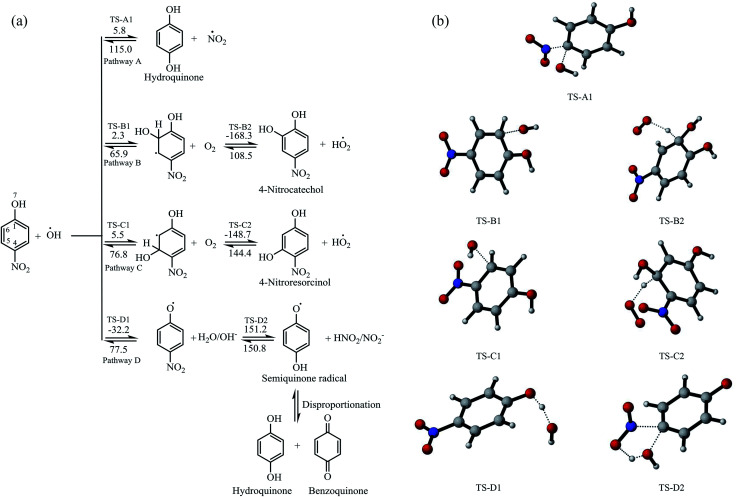
(a) Oxidative degradation reaction pathways for the hydroxyl attack and hydrogen abstraction on 4-nitrophenol to give 4-nitrocatechol, 4-nitroresorcinol and semiquinone radical which eventually disproportionate into hydroquinone and benzoquinone (activation energies (numbers on arrows) for the forward and backward reactions are given in kJ mol^−1^). (b) Optimized geometries of the transition states.

The degradation reaction mechanisms of the oxidation of 4-nitrophenol were studied using the B3LYP/6-31G(d) method in the gas phase based on the reaction between 4-nitrophenol and hydroxyl radical.^38,39^ The reaction pathways for the attack at different positions of 4-nitrophenol by hydroxyl radical were considered and these are represented in Fig. 6a. The optimized geometries of the transition states are illustrated in Fig. 6b. The intrinsic reaction coordinates are provided as ESI in Fig. S2† is found from previous reports that molecular oxygen which is evolved at the cathode in an electrochemical system plays an important role in the degradation process and therefore, subsequent reactions with molecular oxygen were also investigated.^66,67^

It was found that the reaction of hydroxyl radical with the 4-nitrophenol has low activation energy and pathway D is barrierless. The addition of hydroxyl radical at position C-4 leads to the formation of hydroquinone and NO_2_ radical, having activation energy of 5.8 kJ mol^−1^ for the forward reaction and 115.0 kJ mol^−1^ for the reverse reaction (Fig. 6a).

**Fig. 7:**
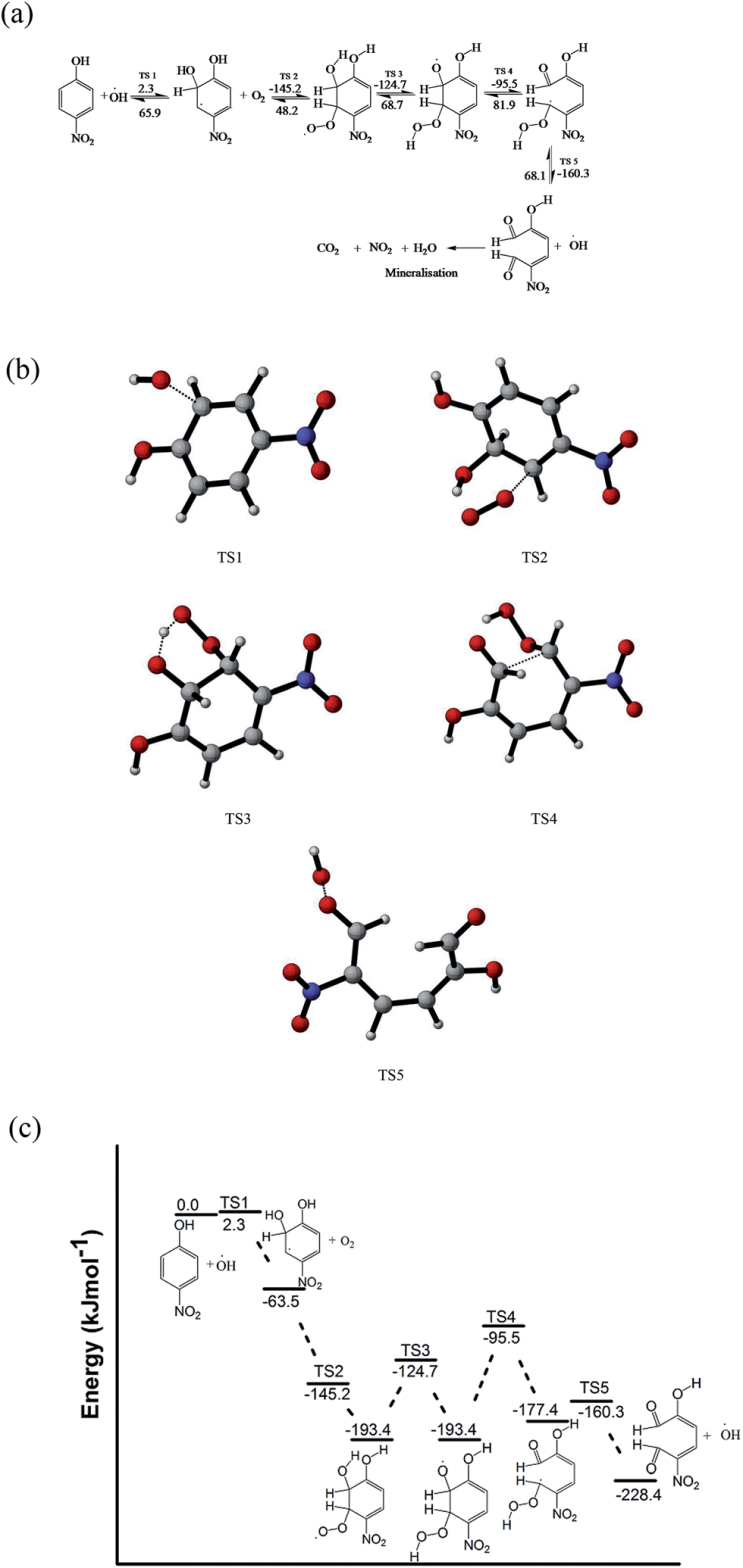
(a) Plausible mechanism for the 4-nitrophenol oxidative degradation reaction leading to the ring opening of the molecule (activation energies (numbers on arrows) for the forward and backward reactions are given in kJ mol^−1^, the activation energies of TS2–TS5 were calculated with respect to the 4-nitrocatechol radical and O_2_ as reactants). (b) Transition states at various steps of the ring opening of 4-nitrophenol. (c) Energy profile for the ring opening reactions.

The pathways (B and C) of the hydroxyl radical attack on the *ortho* and *meta* positions of 4-nitrophenol have low activation energies of 2.3 and 5.5 kJ mol^−1^, respectively. Subsequently, the substitution reaction was accompanied by proton abstraction by molecular oxygen produced in the electrochemical system/solution. This leads to the production of 4-nitrocatechol, 4-nitroresorcinol and hydroperoxyl radical (HO_2_˙). 4-Nitrocatechol and 4-nitroresorcinol are some of the oxidation products of 4-nitrophenol reported in literature, where 4-nitrocatechol is preferentially formed as this pathway is kinetically favoured.^68–71^ These results are also in agreement with the products obtained for the computational studies for the degradation of 4-chlorophenol leading to 4-chlorocatechol and hydroperoxyl radical.^66^ In addition, the molecular oxygen can also react with the *ortho* OH adduct to form peroxyl radicals and eventually, leads to the ring opening and release hydroperoxyl radical (Fig. 7). The ring opening gives rise to intermediates that are oxidised by OH radical and mineralised into carbon dioxide, water and nitrates.^66,70^

Another possible degradation pathway is the abstraction of proton from the hydroxyl group on 4-nitrophenol (pathway D). This pathway is found barrierless and can occur spontaneously, leading to the production of 4-nitrophenoxyl radical and water. The nitrophenoxyl radical and water further reacts to generate a semiquinone radical which was found to be endothermic and slow due to the large activation energy computed (151.2 kJ mol^−1^). Semiquinone radicals are generally known to break down into hydroquinone and 1,4-benzoquinone which are other oxidation products of 4-nitrophenol which were determined in several experimental studies.^66,68–70,72^ The energy profile for the pathway A is found in the supplementary information Fig. S3.†

It can be deduced from the DFT study that for the photoelectrocatalytic degradation of 4-nitrophenol by hydroxyl radical generated in the system led to the formation of benzoquinone, hydroquinone, 4-nitrocatechol and 4-nitroresorcinol. Reaction with the *ortho* OH adduct can also cause ring opening which would be further oxidised or mineralised to less harmful products. In addition, there is generation of hydroperoxyl radical and hydroxyl radical in the system which can initiate more attacks.

## 4 Conclusion

In summary, Pd–ZnO-EG electrode was constructed from a Pd–ZnO-EG nanocomposite, synthesised by a hydrothermal method and characterised using various techniques. The Pd–ZnO-EG nanomaterials have large surface area, pore size and volume, and strong absorption in the visible light region. The electrode was used for the photoelectrocatalytic removal of 4-nitrophenol as a target water pollutant. The Pd–ZnO-EG electrode showed improved photoelectrocatalytic activity in relation to ZnO-EG and EG electrodes for the removal of the 4-nitrophenol. This is as a result of the Pd nanoparticles and the conducting EG acting as collectors for the photoexcited electrons, thereby aiding charge transfer and reducing recombination of charges. In addition, the photocurrent responses showed that the Pd–ZnO-EG nanocomposite electrode could be employed as a good photo-electrode for the benefits of photoelectrocatalytic processes and environmental remediation such as treatment of industrial wastewaters. The oxidative degradation of 4-nitrophenol by hydroxyl radical was predicted using DFT method which led to the generation of hydroquinone, benzoquinone, 4-nitrocatechol, 4-nitroresorcinol and the ring opening of the 4-nitrophenol. This was obtained by DFT computations of plausible reaction mechanism and pathways resulting from hydroxyl radical attacks on different positions on the molecule of 4-nitrophenol, accompanied by hydrogen abstraction by ground state oxygen reactions. Furthermore, hydroxyl radical was regenerated which can further oxidised the ring structure and initiates a new degradation process.

## Conflicts of interest

There are no conflicts of interest to declare.

## Supplementary Material

RA-008-C8RA00180D-s001
