# Supplementary material for: Photoelectrocatalytic application of palladium decorated zinc oxide-expanded graphite electrode for the removal of 4-nitrophenol: experimental and computational studies
Source: RSC Adv. 2018 Mar 13;8(19):10255–66. doi: 10.1039/c8ra00180d (PMC9078825; doi:10.1039/c8ra00180d)
Supplement: RA-008-C8RA00180D-s001 [file RA-008-C8RA00180D-s001.pdf]

Electronic Supplementary Material (ESI) for RSC Advances.
This journal is © The Royal Society of Chemistry 2018

Supplementary information

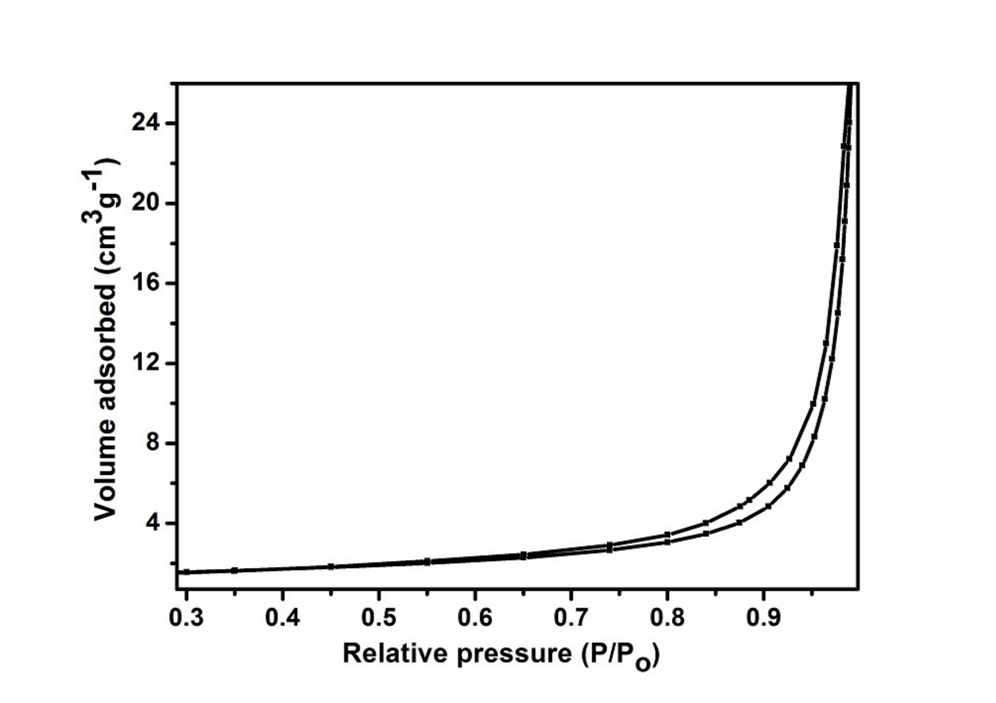

Figure S1. Nitrogen adsorption-desorption isotherms of Pd-ZnO-EG sample.

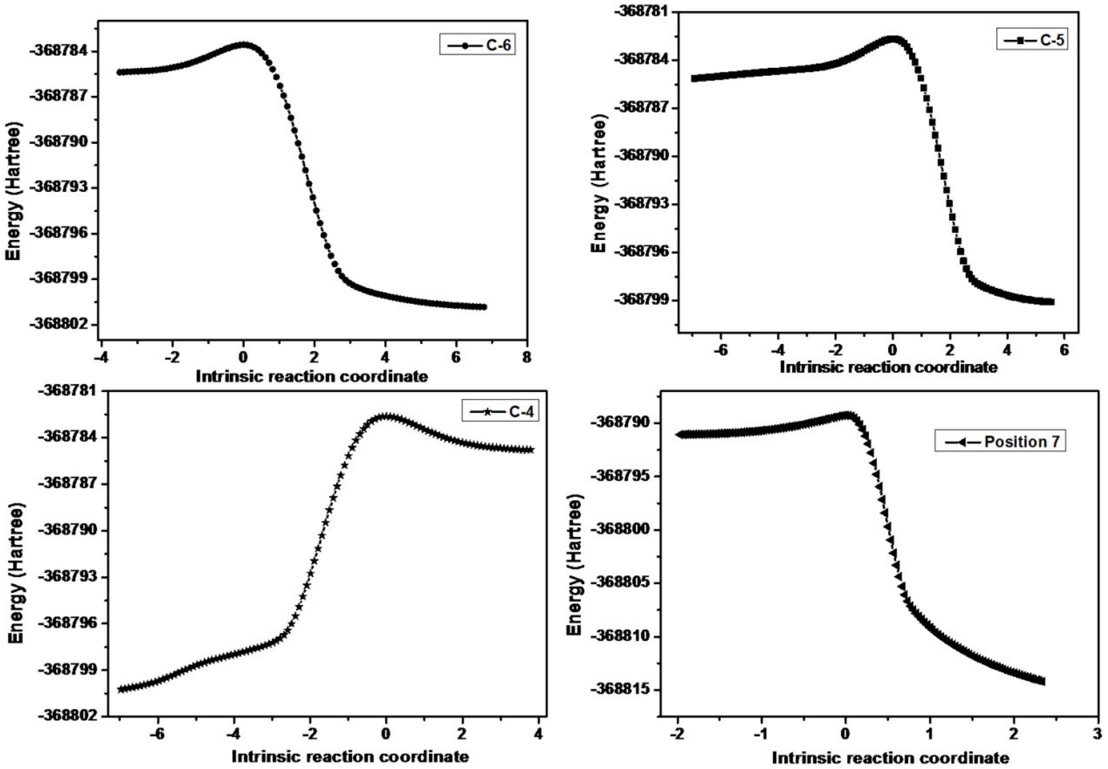

C-6
C-5
C-4
Position 7
Energy (Hartree)
Intrinsic reaction coordinate

a)

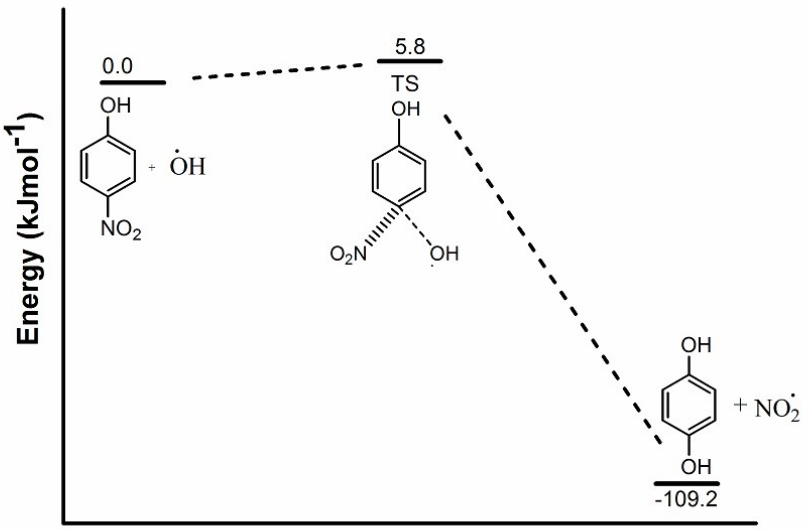

Energy (kJmol-1)
0.0
5.8
TS
-109.2
OH
NO2
OH
O2N
OH
OH
OH
+ NO2

b)

Figure S2. (a) Various reaction coordinates for the hydroxyl attacks on the various positions of the 4-nitrophenol. (b) Energy profile diagram for the nitrophenol degradation pathways leading to the formation hydroquinone from the hydroxyl attack on position 4.

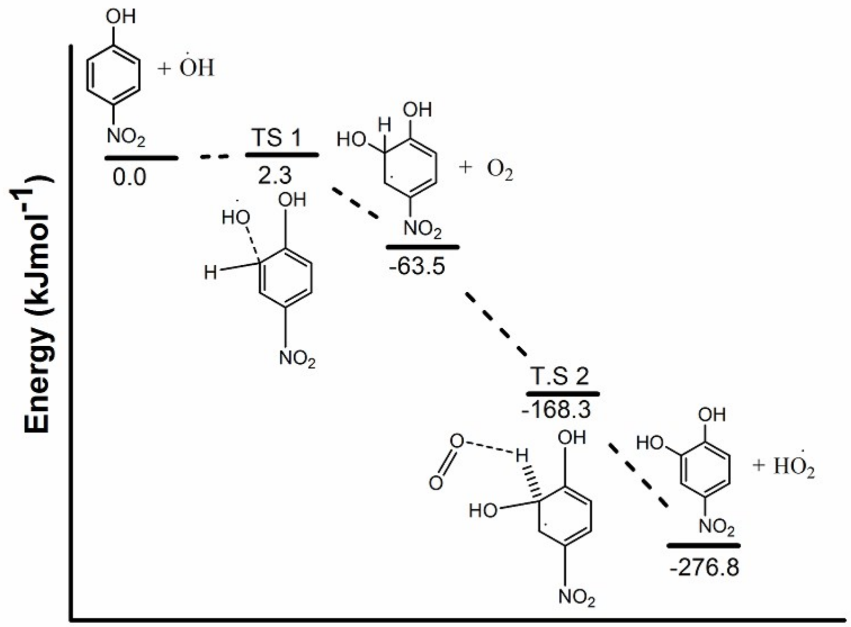

a)

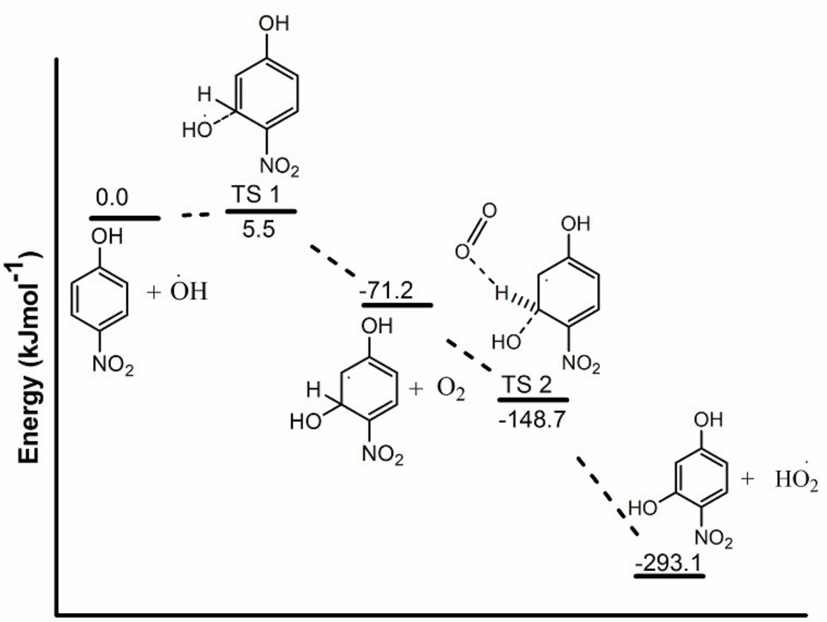

Energy (kJmol-1)
0.0
TS 1
5.5
-71.2
TS 2
-148.7
-293.1
+ ȮH
+ O2
+ HȮ2
OH
NO2
HO
H

b)

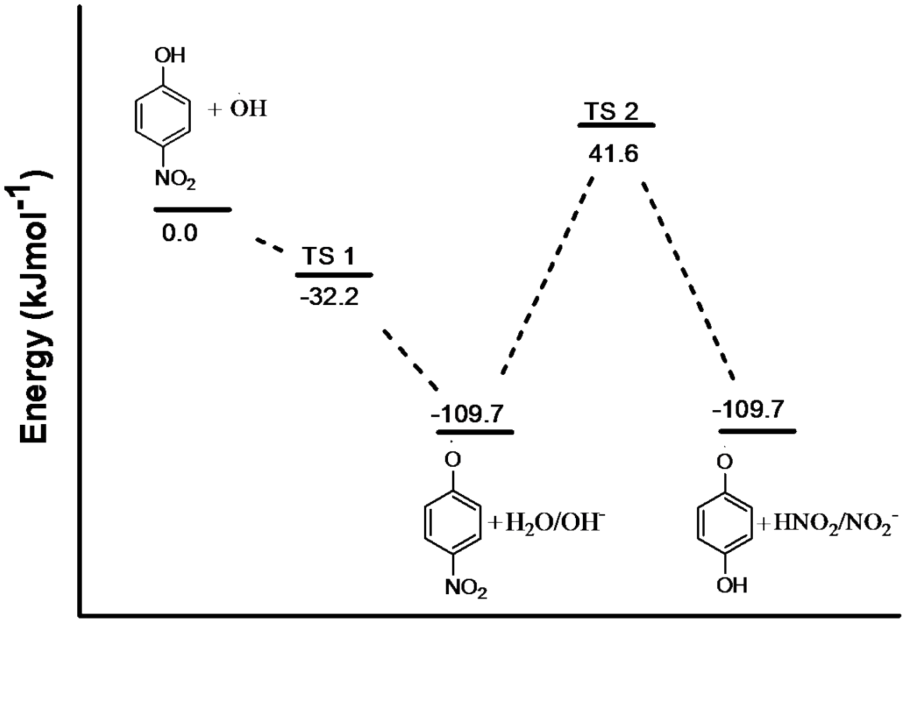

Energy (kJmol-1)
+ ȮH
0.0
TS 1
-32.2
TS 2
41.6
-109.7
-109.7
+H2O/OH-
+HNO2/NO2-
OH
NO2

c)

Figure S3. Energy profile for the 4-nitrophenol degradation pathways leading to the formation of (a) catechol, (b) resorcinol, and (c) semiquinone radical.
